# SOP: antibody-associated autoimmune encephalitis

**DOI:** 10.1186/s42466-019-0048-7

**Published:** 2020-01-15

**Authors:** Rosa Rössling, Harald Prüss

**Affiliations:** 1 Department of Neurology and Experimental Neurology, Charité – Universitätsmedizin Berlin, CharitéCrossOver, R 4-334 ,Charitéplatz 1, 10117 Berlin, Germany; 2 German Center for Neurodegenerative Diseases (DZNE) Berlin, Berlin, Germany

**Keywords:** Autoimmune, Encephalitis, Limbic encephalitis, Antibody, Paraneoplastic, NMDAR

## Abstract

**Background:**

Antibody-mediated and paraneoplastic autoimmune encephalitides (AE) present with a broad spectrum of clinical symptoms. They often lead to progressing inflammatory changes of the central nervous system with subacute onset and can cause persistent brain damage. Thus, to promptly start the appropriate and AE-specific therapy, recognition of symptoms, initiation of relevant antibody diagnostics and confirmation of the clinical diagnosis are crucial, in particular as the diseases are relatively rare.

**Aim:**

This standard operating procedure (SOP) should draw attention to the clinical presentation of AE, support the diagnostic approach to patients with suspected AE and guide through the necessary steps including therapeutic decisions, tumour screening and exclusion of differential diagnoses.

**Method:**

Based on existing diagnostic algorithms, treatment recommendations and personal experiences, this SOP gives an overview of clinical presentation, diagnostic procedures and therapy in AE. Additional information is provided within an accompanying text and a table describing the most important autoantibodies and their characteristics.

**Results:**

The initial steps of the AE flow chart are based on clinical symptoms and the patient’s history. Assignment to paraneoplastic or antibody-mediated AE is sometimes clinically possible. Diagnostics should include MRI, EEG and CSF analysis with antibody panel diagnostic. Definite AE can be diagnosed if the underlying antibody is compatible with the clinical presentation. Classification of probable AE may be possible even with negative anti-neuronal autoantibodies if the clinical presentation and laboratory abnormalities are highly suggestive of AE. The confirmed AE diagnosis requires immediate initiation of immunotherapy.

**Conclusion:**

The SOP facilitates the recognition of patients with AE and presents the necessary diagnostic and therapeutic steps.

## Introduction

Autoimmune encephalitis (AE) is an often rapidly progressive inflammatory neurological disease with subacute onset. The best characterized and most common form of AE is anti-NMDA receptor (NMDAR) encephalitis, defined by cerebrospinal fluid (CSF) IgG antibodies targeting the NMDA type glutamate receptor. Patients can present with altered mental status, reduced levels of consciousness, deficits in working/short-term memory that develop in usually less than 3 months (frequently within < 6 weeks), and may show psychosis or new epileptic seizures [1].

AE comprises antibody-mediated and paraneoplastic (i.e. usually cytotoxic T-cell-mediated) encephalitides, which form a heterogeneous group of autoimmune neuropsychiatric diseases with still broadening clinical phenotypes and numerous novel autoantibodies identified in recent years (Table 1). The incidence is estimated at 5–10 per 100.000 inhabitants per year [1] with often specific age and gender preferences for a given antibody.

**Table 1:** Most important antibodies and clinical syndromes

Antigen	Characteristics	Preferred detection	Age/Gender	Tumour
Antibodies against neurotransmitter receptors [2]				
NMDAR [3]	Schizophreniform psychosis, perioral dyskinesia, epileptic seizures, coma, dystonia, hypoventilation; cMRI frequently normal, often CSF pleocytosis, EEG with slow waves, can show extreme delta brush	CSF	Most prevalent subtype of AE; All ages, peak in childhood and youth, 75% women	Ovarian teratoma
GABAaR	Epileptic seizures, schizophreniform syndrome, refractory status epilepticus and epilepsia partialis continua	Serum or CSF	Younger adults; m > f (1.5:1)	Hodgkin lymphoma
GABAbR	LE with frequent epileptic seizures	CSF	Older adults f = m	50% lung cancer (SCLC)
AMPAR	LE, Epileptic seizures, memory deficits, psychosis; CSF often normal	CSF	Older Adults f > m (2.3:1)	In 70% lung/ breast cancer
mGluR5	LE, Ophelia syndrome (depression, agitation, hallucination, memory deficits, personality changes)	CSF	Young adults, m > f, (1.5:1)	Hodgkin lymphoma
GlycinR	PERM (progressive encephalomyelitis with rigidity and myoclonus), SPS, cognitive deficits	Serum or CSF	Older adults f = m	Thymoma (< 10%)
DPPX	LE with tremor, myoclonus, hallucinations, therapy refractory diarrhoea	CSF	Older adults f < m (1:2.3)	Not known
Antibodies against ion channel subunits or cell adhesion molecules [4, 5]				
LGI1	Facio-brachial dystonic seizures, amnesia, psychosis, LE, Medial temporal lobe hyperintensities in MRI, hyponatremia	Serum	Second most common type of AE; Adults > 40 years, m > f (2:1)	Rare
Caspr2	LE, neuromyotonia, Morvan syndrome, can slowly progress over up to 1 year; similar to LGI1, but no hyponatremia	Serum	Elderly m > f (9:1)	Thymoma possible
IgLON5	REM- and non-REM sleep disorders, sleep apnoea, stridor, dysarthria, dysphagia, dysautonomia, movement disorders, dementia	Serum	Older adults, f = m	Not known
Antibodies against glial structures				
GFAP [6]	Headache, subacute encephalopathy, optic papillitis, myelitis, CS	Serum and CSF	f = m	Possible
Antibodies against Intracellular (onconeural) antigens [7, 8]				
Hu (ANNA-1)	Encephalomyelitis, brainstem encephalitis, LE, Denny-Brown syndrome	Serum	Large variability, depending on tumour occurrence	> 90%, SCLC
Ri (ANNA-2)	OMS, CS, encephalomyelitis	Serum		> 90%, Ovary, breast cancer
Yo (PCA-1)	CS	Serum		> 90%, Ovary cancer
Ma2	LE, CS, diencephalic/ hypothalamic involvement	Serum		> 90%, Testicular, lung cancer
CV2 (CRMP5)	Encephalomyelitis, LE, CS	Serum		> 90%, SCLC, thymoma
Amphiphysin	SPS	Serum		> 90%, Breast, SCLC
GAD	SPS, LE, ataxia	Serum and CSF	Middle aged, f > m (4:1)	Only rarely associated with tumour

LE: limbic encephalitis, SPS: Stiff-person syndrome, OMS: Opsoclonus-myoclonus syndrome, CS: cerebellar syndrome, SCLC: small cell lung cancer, PCD: paraneoplastic cerebellar degeneration

**Fig. 1:**
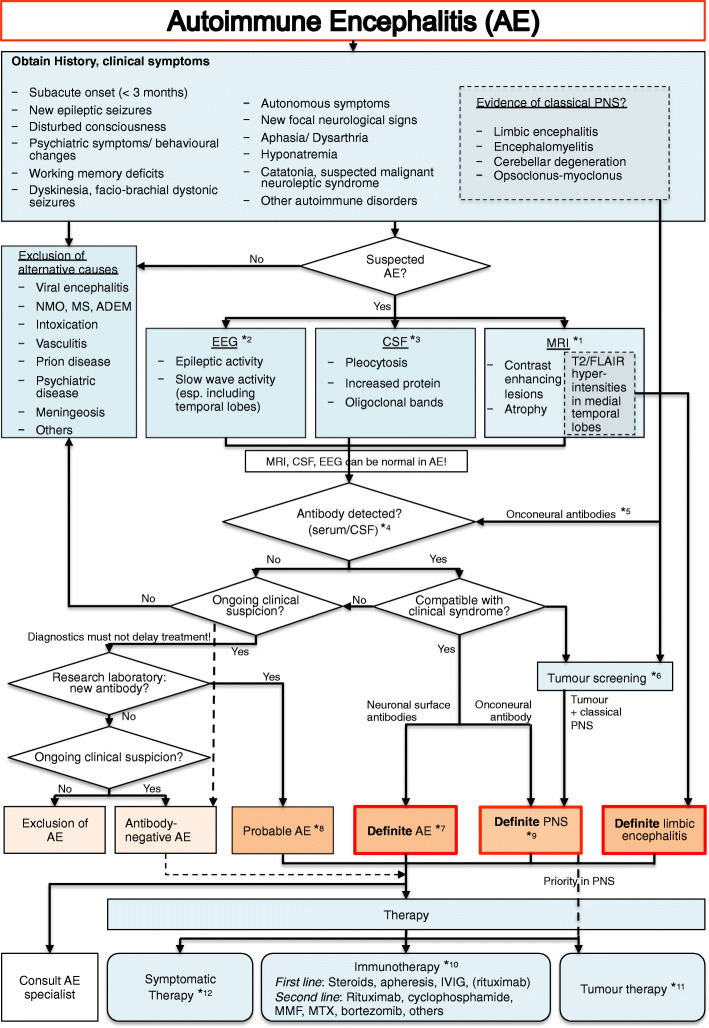
Flow chart for the diagnosis of suspected autoimmune encephalitis. AE: Autoimmune encephalitis, PNS: paraneoplastic neurological syndrome, CSF: cerebrospinal fluid, FLAIR: fluid attenuated inversion recovery, MTX: methotrexate, MMF: mycophenolat mofetil, IVIG: intravenous immunoglobulin, NMO: neuromyelitis optica, MS: multiple sclerosis, ADEM: acute disseminated encephalomyelitis

Although much has been learned about AEs and their variable clinical phenotypes, diagnosis is often still delayed, partly related to incomplete awareness and false assignment of the presented symptoms [9]. Early diagnosis, however, is crucial in order to promptly start the appropriate therapy which also depends on the type of AE. This SOP aims for facilitating the approach to patients with central nervous system symptoms suspicious of AE, for raising awareness to the clinical presentation of such patients, and for proposing the necessary diagnostic and therapeutic steps (Fig. 1).

Antibodies in antibody-mediated AE are mostly directed against neuronal surface antigens. By binding to excitatory transmitter receptors (NMDA, AMPA), inhibitory transmitter receptors (GABAb, GABAa, glycine), ion channel subunits and cell adhesion molecules (CASPR2, IgLON5) or soluble synaptic proteins (LGI1) they are directly pathogenic. Autoantibody formation can be triggered by viral or tumour exposition [10], but mechanisms are unknown in most cases. In contrast, antibodies in paraneoplastic neurological syndromes (PNS) bind to intracellular antigens and therefore cannot cause the disease directly, even though they serve as valuable biomarkers for an underlying tumour (such as Hu, Ri, Yo or Ma2 antibodies). Neuronal damage in these cases is rather caused by cytotoxic T-cells with oligoclonal T-cell receptor expansion and autoreactivity against neuronal structures. Triggers for antibody production in PNS are presumably ectopically presented antigens on the tumour cells that are otherwise exclusively expressed in neurons. Parenchymal invasion of immune cells leads to early neuronal cell damage with consecutive cell death. Among the antibodies targeting intracellular antigens, GAD and amphiphysin antibodies are an exception as they seem to be pathogenically relevant despite their intracellular antigen location. Furthermore, GAD antibodies are only rarely associated with a tumour [11].

### Diagnosis

Early diagnosis of antibody-mediated AE or PNS can be difficult because of the broad spectrum of disease manifestations. Clinical presentation is regularly not limited to a well-defined syndrome. However, there are some *red flags* that strongly indicate AE. These include common signs and symptoms, such as altered level of consciousness, dyskinesia, faciobrachial dystonic seizures, autonomic dysfunction, focal neurological signs, aphasia/dysarthria, hyponatremia, headache, catatonia or suspected malignant neuroleptic syndrome. In PNS every level of the nervous system can be affected. In this SOP, we focus on classical central nervous system presentations that include encephalomyelitis, limbic encephalitis, subacute cerebellar degeneration and opsoclonus-myoclonus syndrome. Additional autoimmune disorders in the patient’s history (such as thyroid disease, diabetes, vitiligo or inflammatory bowel disease) suggest increased susceptibility to autoimmunity and should likewise prompt antibody testing even in the absence of abnormalities in MRI, EEG or CSF (Fig. 1).

*1 MRI is a central part of the standard work-up in AE, even though imaging might only show non-specific changes in early stages of disease. For example, in NMDAR encephalitis, MRI is unremarkable in more than 50% of patients despite severe clinical symptoms. Similarly, at the beginning of clinical symptoms in paraneoplastic cerebellar degeneration (PCD), imaging might be normal and cerebellar atrophy is only visible later in the disease course. On the other hand, increased signal in T2-weighted/FLAIR imaging in the medial temporal lobes allows the diagnosis of ‘definite limbic encephalitis’ in the appropriate clinical context [1]. MRI in patients with GABAaR encephalitis is almost always abnormal with multifocal diffuse cortical and subcortical T2/FLAIR hyperintensities. Severity of MR-morphological damage can correspond with prognosis. For instance, in LGI1 encephalitis bilateral hippocampal atrophy indicates poor outcome with persistent cognitive deficits. Furthermore, atrophy might progress during follow-up, even if prior imaging was unremarkable [12].

*2 EEG is also very helpful in PNS and antibody-mediated AE. Although it is only rarely specific, EEG is altered in most patients and often shows general slowing or helps to detect subclinical seizures or a non-convulsive status epilepticus. In patients with NMDAR encephalitis, an ‘extreme delta brush’ is a rare but specific finding that can lead to diagnosis [13].

*3 CSF analysis is always recommended in the workup of patients with suspected AE. Basic CSF parameters (white blood cells, protein) differ profoundly depending on the underlying type of AE. Inflammatory changes in the CSF are typically seen in patients with antibodies against NMDAR, GABAbR, AMPAR or DPPX, whereas antibodies targeting CASPR2, LGI1, GABAaR or glycine-R can be associated with normal CSF findings. CSF from IgLON5 antibody-positive patients typically shows increased protein. Oligoclonal bands occur mostly with antibodies associated with pleocytosis, an exception is GAD encephalitis, where oligoclonal bands without further CSF findings are typical [14]. For antibodies that are predominantly found in the CSF (such as NMDAR antibodies), titres are not only relevant for diagnostics, but also helpful during follow-up. In 14–20% of patients with anti-NMDAR encephalitis antibodies are present in CSF only. In contrast, serum levels of most antibodies do not correlate well with clinical course and prognosis. For some of them, antibody titres should reach certain levels to support the diagnosis. For example, serum Caspr2 antibodies < 1:320 or GAD antibodies < 2000 U/ml are mostly not sufficient to confirm the diagnosis of AE.

**Fig. 2:**
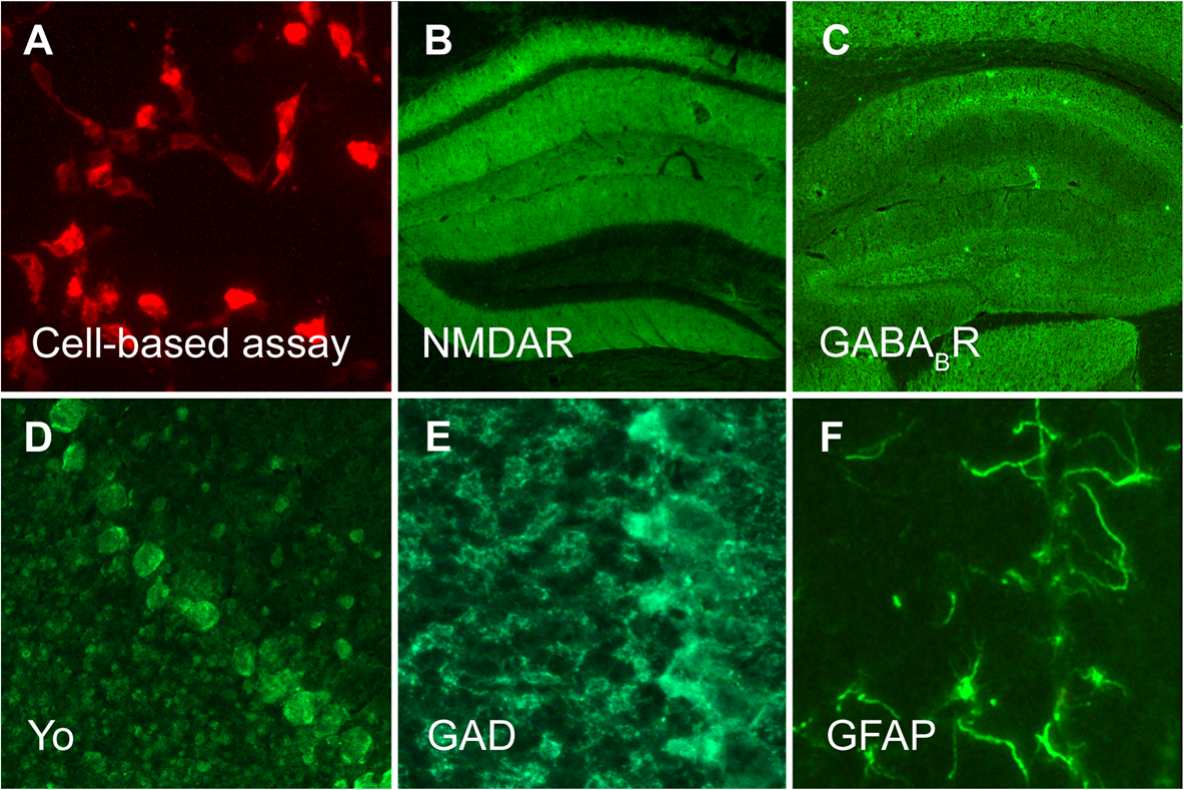
Detection of anti-neuronal autoantibodies for the diagnosis of autoimmune encephalitis. **a** The current gold standard for established surface antibodies is the cell-based assay (CBA), in which diverse target antigens (in this example NMDAR) are recombinantly expressed on the surface of cultured cells. Binding of patient antibodies from CSF or serum samples can be visualized with fluorescent dyes. **b** The same CSF sample of a patient with NMDAR encephalitis shows strong binding on a mouse hippocampus section with the characteristic NMDAR distribution. **c** Autoantibodies to GABAbR also show strong binding to hippocampus tissue, but with a clearly distinguishable pattern. **d** Antibodies to onconeural antigens can be visualized by staining of line blots (not shown) or by their intracellular binding on brain sections, here Yo antibody-positive Purkinje neurons on a mouse cerebellum section. **e** GAD antibodies show a punctate pattern around cerebellar granule cells and Purkinje neurons. **f** Immunohistochemistry using brain sections also allows the detection of antibodies targeting glia cells, such as against GFAP. The methodology further permits detection of as yet undetermined anti-brain antibodies in research laboratories. D and E modified from “Prüss et al. 2017, Neurotransmitter”

*4 In every patient with a history suspicious for AE, a panel of autoantibodies should be analysed (Fig. 2), even if MRI, EEG and basic CSF parameters are normal. In suspected AE it is always recommended to test both serum and CSF to not overlook treatable conditions. Some clinicians test serum first and expand diagnosis to CSF if results were negative, however, this will normally delay diagnosis. Common panels of neuronal surface autoantibodies include detection of IgG against NMDAR, Caspr2, LGI1, GABAbR, AMPAR, followed by GABAaR, mGluR5, glycine-R and IgLON5.

*5 If clinical presentation matches a classical PNS or if a neoplasm is suspected, analysis of onconeural antibodies is mandatory. Panel diagnostics including the following antibodies is recommended: Hu, Ri, Yo, Ma, CV2 and amphiphysin. Non-onconeural antibodies targeting neuronal surface antigens like GABAbR, AMPAR, mGluR5 and NMDAR (with the exception that tumours are typically teratomas) might also show high cancer association.

*6 In PNS, neurological symptoms often manifest weeks to months before recognition of the tumour. The likelihood of a tumour is > 95% when patients present with a classical PNS and a well-characterised onconeural antibody. The antibody itself may give a hint to the underlying tumour, e.g. Yo antibodies found in patients with PCD are characteristically associated with ovarian cancer. Standard imaging in tumour search includes body CT with contrast as well as MRI and ultrasound. If the findings are negative, a whole-body FDG-PET should be performed. At the beginning, tumours in PNS might be too small to be found, but as their detection is essential in providing the appropriate therapy, imaging should be repeated every 6 months for a minimum of three years [8].

*7 With confirmation of a specific antibody that is in line with the clinical syndrome, the diagnosis of ‘definite AE’ can be made. Examples include the presence of CSF NMDAR antibodies in a young woman with schizophreniform psychosis, epileptic seizures and hypoventilation, the presence of GABAaR antibodies in a middle-aged man with new onset epilepsy progressing to status epilepticus, or the detection of LGI1 antibodies in an older adult with new-onset amnesia, hyponatremia and marked behavioural changes. Immediate immunotherapy is required in almost all cases with ‘definite AE’.

*8 We propose the diagnosis of ‘probable AE’ also in clinical constellations that are supported by the finding of a novel neuronal surface autoantibody in a research laboratory, using immunohistochemistry on brain sections (Fig. 2) or staining of live neurons. We generally recommend the CSF (+/− serum) analysis of unclear cases on a research basis if commercial antibody assays show negative results. Importantly, treatment should be initiated similar to definite AE and must not be delayed by an extended antibody search. The diagnosis of ‘antibody-negative AE’ can be made in the absence of an anti-neuronal antibody if findings from clinical presentation, MRI or CSF strongly suggest an autoimmune aetiology and after exclusion of differential diagnoses [1].

*9 Diagnosis of ‘definite PNS’ according to Graus et al. [7] can be made in clinical constellations of AE where [1] the classical PNS and cancer develop within five years of the PNS diagnosis or [9] the neurological syndrome is associated with well-characterised onconeural antibodies (anti-Hu, Yo, CV2, Ri, Ma2 or amphiphysin), even in the absence of cancer.

### Therapy

It is the nature of AE that brain dysfunction frequently leads to psychiatric symptoms with patient’s rejection of consent for immediate diagnostics and treatment, comparable to infectious encephalitis. This is particularly important for clinical practice as delayed treatment of AE will result in irreversible damage and as the relative novelty of AE often leads to uncertainty about the patient’s expressed will in clinical practice. We recently suggested an approach for AE patients with the lack of ability to give consent, but treatment is medically reasonable or even demanded [15].

*10 Therapy depends on the clinical syndrome and the underlying antibody. In patients with AE caused by antibodies against neuronal surface antigens, immunotherapy is usually more successful than in patients with antibodies targeting intracellular proteins. Treatment studies in patients with AE are sparse and focus on the most common forms of AE, such as NMDAR encephalitis. Although early therapy is critical, marked recovery can be seen in some patients with antibody-mediated AE in whom therapy is only started months after disease onset. First-line therapy in antibody-mediated AE comprises high-dose intravenous methylprednisolone (1000 mg/d i.v. for 5 days), therapeutic apheresis (at least 5 times every other day, in cases with predominant CSF antibodies usually 7–10 treatments needed) or intravenous immunoglobulins (2 g/kg body weight over 3–5 days). If no treatment effect is seen after two weeks, second-line therapy should be started with no delay. The anti-CD20 antibody rituximab is frequently used (1000 mg, with the first two administrations at day 1 and day 15 followed by 6 months intervals). Due to its good tolerance and efficacy, many centres use rituximab as first-line therapy in AE patients with surface autoantibodies. Steroids might be sufficient in patients with LGI1 antibodies, but clearly not in patients with NMDAR encephalitis who should receive rituximab also to prevent relapses. If high antibody titres persist parallel to clinical symptoms, repeated apheresis should be considered. Antibody-mediated AE can be monophasic, i.e. maintenance treatments can often be stopped after 1–3 years. Comparative studies of the respective therapeutic option are still lacking. In any case, early initiation of immunotherapy is crucial not only regarding the acute phase of the disease, but also for long-term outcome. As shown in patients with NMDAR encephalitis, long-term outcome might be impaired by persistent cognitive deficits [16].

Cyclophosphamide is another option for second-line therapy and might be combined with rituximab. Many other treatments have been used with variable success, including mycophenolat mofetil, methotrexate or azathioprine. Promising new data suggest that the proteasome inhibitor bortezomib might be a valuable option in patients with surface antibody-mediated AE [17]. Given the ongoing expansion of immunotherapies in these indications (e.g. daratumumab, tocilizumab or autologous stem cell transplantation) and the right of all patients with rare AE to potentially receive such treatments, *consultation of a specialised AE centre is generally recommended* after confirmation of diagnosis.

In paraneoplastic AE with antibodies targeting intracellular proteins, rituximab, intravenous immunoglobulins and therapeutic apheresis often have only little effect as the antibodies are not directly pathogenic, but neuronal damage is caused by cytotoxic T-cells. Furthermore, therapy in PNS is often delayed and substantial irreversible neuronal cell damage has already occurred at the time of presentation. If cell damage is visibly progressing in brain imaging after 3 to 6 months in spite of advanced immunotherapy, discontinuation of therapy is recommended in these patients.

*11 Evidence of a tumour requires, if possible, prompt and complete removal to withdraw the auto-antigen that is ectopically produced on tumour cells and likely triggers the production of autoantibodies. However, neuronal damage will often progress, especially in PNS [7]. Please see Table 1 for common associations of an antibody with a specific tumour.

*12 Symptomatic therapy depends on the form of AE. Antiepileptic therapy is frequently required as AE commonly leads to epileptic seizures. Antiepileptic drugs should be tapered after the encephalitic phase given that in surface antibody-mediated AE seizures are mainly acute-symptomatic. Psychotic symptoms often require transient treatment with antipsychotic drugs, which might also be tapered after the initial disease phase. With status epilepticus, autonomous symptoms or major behavioural abnormalities, patients regularly require intensive care unit treatment including sedation and mechanical ventilation. Physiotherapy and speech therapy can further help to improve the outcome.

## Conclusion

In patients with suspected antibody-associated AE, it is essential to analyse the patient’s history for the above-mentioned *red flags*. Standard diagnostic work-up includes EEG, MRI, CSF analysis and testing for anti-neuronal autoantibodies. ‘Definite AE’ or ‘definite PNS’ can be diagnosed when a detected antibody is compatible with the clinical syndrome. Treatment should be initiated as soon as possible and must not await pending antibody analysis. Consultation of an AE specialist is generally recommended after confirmation of the diagnosis of AE.

## Data Availability

Available to readers on request. The authors declare that they have no competing interests.
